# Natural and commercial antibiotic comparison with drugs modeling cell integrity cell stability of Bio-Kinetics changes under morphological topographies cells with lower toxicological characteristics for multidrug resistances problem

**DOI:** 10.1016/j.sjbs.2022.103351

**Published:** 2022-07-01

**Authors:** Waseem Ahmed, Rafia Azmat, Nabila Chendouh-Brahmi, Rasheed Ahmed, Saima Naz, Abdul Qayyum, Ahmad El Askary, Amal F. Gharib, Amani A. Alrehaili, Nausad Ali

**Affiliations:** a Department of Horticulture, The University of Haripur, Pakistan; b Department of Chemistry, University of Karachi, Pakistan; c Laboratory of 3BS, Faculty of Life and Nature Sciences, University of Bejaia, 06000 Bejaia, Algeria; d Department of Soil Science, The University of Haripur, Pakistan; e Division of Science and Technology Department of Chemistry, University of Education Lahore, Multan Campus, Pakistan; f Department of Agronomy, The University of Haripur, Pakistan; g Department of Clinical Laboratory Sciences, College of Applied Medical Sciences, Taif University, Taif 21944, Saudi Arabia; h Department of Plant Breeding & Genetics, The University of Haripur, Pakistan

**Keywords:** Compartment, Herbal, Medicines, Pharmacokinetics, Cytotoxicity, Antibacterial resistant

## Abstract

Antibacterial drug-resistant strains are a serious problem of bacterial treatments nowadays and have a concern. The plant exacts of *Adhatoda vasica* and *Calotropis procera* are well-known for their role as antibiotic agents. The extraction of novel antibiotic compounds was done by HPLC-DAD, their yield is quantified by numerous solvents. The complete biological activity with antioxidants, bio-kinematicof four compounds of B-Sitosteryl linoleate, Myristyl diglucoside, D-Triglucopyranoside, and S- allylcysteine acids were studied. The supercritical fluid extraction techniques were the best strategies for higher yield, accuracy clarity, and inter, intra process of all four compounds. *A. vasica* and *C. procera* samples and investigated in six different solvents. D-Triglucopyranoside (13.81 ± 0.48%), Myristyl diglucoside (11.81 ± 0.41%), B- Sitosteryl linoleate (12.81 ± 0.48%), and s-allylcysteine acids (14.81 ± 0.31%) were higher. The design and action of compounds were applied to proper compartmental pharmacokinetic modelling for in-depth design understanding. The morphology and structure of bacterial cells with the extracted compounds upheld the permeability of cell membranes, membrane integrity, and membrane potential and lower the bacterial binding capacity the infectious index was measured in transmission electron microscopy (TEM) and their alteration process. Plants have well upheld the cellular permeability The toxicity test was performed on both extracted samples with concentrations (1, 0.4, and 0.8%). The areas under plasma half-life of compounds with their solubility, abortion level were higher in four compounds showed the potential of novel antibiotics. The novel medicinal plants used as antibiotics could be the best sources of infection control as a source of future medicines with antibacterial potential solving multidrug issues of bacteria in the world.

## 1 Introduction

Medicinal plants are safe and feature sources of various agents as widely used in medicines and drugs which have significant role in pharmaceutical industries, and a source of powerful antioxidants that were utilized in synthetic dyes and minimize the oxidative stress of the body (Chhikara et al., 2019, Mani and Kumar, 2014). Advanced techniques contribute a basic role to isolation of novel compounds for drug preparation for the treatment of chronic diseases in humans (Quideau et al., 2011). Recently, environmental stress and pollutants in soil and water are a concern for spreading such diseases that are threat to human threat to human health. (Vij and Prashar, 2015). The commercial antibiotic is phenoxymethylpenicillin. dicloxacillin, amoxicillin with clavulanic acid ampicillin, in natural antibiotic are various plants extracts same like our plant's apple cider, ginger. Clove, and grapefruit extracted are used as antibiotic here in this study we can isolate the targeted compounds as a natural antibiotic. The constant emission in ecology causes a buildup of residues, which may show resistance in living organisms, resulting in bacterial resistance; as a result, the living cell dies suddenly with such attacks (Mani and Kumar, 2014). Pharmacy and pharmaceuticals have to find the solution as many of the microorganisms have developed antibiotic resistance. The plant-derived antioxidants, such as polyphenols are recognized as a critical component due to their potential useful capabilities in the regulation of lethal compounds of human bodies (Quideau et al., 2011). The medicinal plants include bark, fruits, leaves, roots and seeds could be used in the cure of different human ailments due to their high antioxidant activities (Vij and Prashar, 2015). The novel herbal compounds were b-Sitosteryl linoleate and Myristyl diglucoside revealing a huge spectrum of ability to combat bacterial organisms that cause disorders in humans (Brand, 2012, Cakir et al., 2004). Phenylalanine, a chemical component of Myristyl diglucoside, is derived from plant parts and used to treat a variety of chronic disorders (Alipieva et al., 2014). The Myristyl glucoside seems to have a wide spectrum of biological action even against impacts of many bacterial forms that cause considerable illness in the kidneys, stomach, liver, and digestive tracts in the human body (Pan et al., 2003). These distinct chemicals inhibited the bacterial growth in the human body (Pan et al., 2003). D-Triglucopyranosid seems to be a stronger antibacterial resistant compound that come from plants and has both phenolic and acrylic functional groups, it is isolated from plants and can protect DNA from the degradation (Li et al., 2005). Likewise, polyphenolic groups are used to control the losses of cellular components (Agar and Cankaya, 2020). There is a new class of phenylpropanoid glycosides that regulate a wide spectrum of enzyme activity in humans (Agar and Cankaya, 2020). A review of the literature demonstrated that harmful components or toxins are detoxified through the active consumption of bioactive compounds found in herbal plants (Watson and Preedy, 2016) these compounds engage in several metabolic processes at the molecular level, increasing enzyme changes that involve improvement of the immune system (Jana and Mandlekar, 2009). During the last few years, S-allyl cysteine acid and triglucopyranoside molecules received a lot of interest, which have a wide spectrum of biological functions counting for anti-microbial, antioxidant, anti-carcinogenic, anti-inflammatory, vision enhancement and apoptosis activation as a neuroprotective strategy in humans (Ahmad et al., 1995, Aligiannis et al., 2003, Fuji et al., 2018). The triglucopyranoside functions as an antioxidant removing free radicals and decreasing cell stress reactions by up-regulating cytoprotective mechanisms with potent antimicrobial effects against both positive and negative pathogens (Cook et al., 1993). Due to their redox activity, all chemicals operate as hydrogen donors and singlet oxygen quenchers (Cook et al., 1993, Dimberg et al., 1993, Gulluce et al., 2006). Negative bacteria pose a global threat because of their double cell membrane morphological structure, which allows the infection to enter cells directly as well as cause difficulties in the human body (Kroon and Williamson, 1999). The dissociation, filtration and isolation of vital sections of animals or plants tissues from weak or inactive elements are referred to as the extraction process in pharmaceutics (Morton, 1955, Taylor et al., 2001). The purification procedures and appropriate solvents are an essential part of determining the efficacy and activity of pure solventpotential and its yield (Poole, 2004, Tzschucke et al., 2002, Mustafa and Turner, 2011). Medicinal plants contain a variety of secondary metabolites with antibacterial action inthe treatment of chronic disorders but the solvents and their extraction methods are important (Danlami and Elisha, 2017).

Medical plants contain a variety of health-related chemicals current study approved, four novel compounds with structural properties, as antibiotics of S-allylcysteine acid, D-Triglucopyranoside, B-Sitosteryl linoleate and Myristyldiglucoside were identified in *A. vasica* and *C. procera* through high-performance liquid chromatography with two detectors (DAD-MS/MS). The proper extraction solvents (Ascorbic acid, ethyl acetate, methanol, aqueous, hexane and chloroform), with their efficiency as well as modern extraction techniques microwave-assisted extraction (MAE), cold extraction (CE), liquid–liquid- micro-extraction (LLME), supercritical fluid extraction (SCFE) and exhaustive extraction (EE), had also been used for the separation of the pure compounds were used in future antibiotic The three primary antioxidants activities were tested, and biological performances of extracted plants were applied on the selected gram-positive and negative bacterial pathogen to measure the minimum bacterial concentrations (MBCs), minimum inhibition concentrations (MIC) and combined fractional inhibitory concentration index (CFICI). The compounds were evaluated in a completed designed drugs model under vitro trials with complete bio-efficacy, AUC, changes of bacterial cells responses of permeability of cell membrane, cell membrane integrity, binding activity at cells infectious indexes responses and Acid dissociation constant (pKa). Solubility, absorption capacity following, for the first time compartmental pharmacokinetic modelling was used. The extracted samples and commercial antibiotics were compared in cell morphological changes in the cell of bacterial and altercations changes of bacterial strains were noted in SEM 76 model.

## 2 Experiment

### 2.1 Materials

#### 2.1.1 Chemicals and reagents for experimental trials

Chemicals and reagents which include trolox, ethanol and methanol were obtained by Sigma-Aldrich (Germany). These chemicals were bought from Chroma Dex (USA). The high-quality analytical orthophosphoric acid (89%), tetrahydrofuran, dimethyl sulfoxide (DMSO) and acetone were acquired from Merck (Darmstadt, Germany).

#### 2.1.2 Medical plants collection

*A. vasica and C. procera* matured leaves were harvested from Kanpur, Haripur, Pakistan. The samples were brought into The University of Haripur’s Horticulture Laboratory for biochemical the biochemical research was carried out by using the HPLC and an X Bridge C18 detectors. The plant's repository codes were 771 in *A. vasica* and 1811 in *C. procera*.

### 2.2 Extraction of sample and procedure

The samples of both plants were made by combining pulverized dried leaves (1 g) with carbonated water (10 ml). After that, a mechanized shaker was used to agitate the extract which was then purified via filter paper. The pure solution was maintained in refrigerator after the plant sample was transferred to a separate container (Miller, 1996).

### 2.3 Optimization of various solvents and extraction techniques for novel compounds as efficient yields

The (500 ml) of ethyl acetate, aqueous chloroform hexane, ascorbic acid and methanol were employed to isolate the powdery sample (250 g) out of each plant in room temperature for 48 h. The samples were refined through filter paper. After filtration the samples were vaporized and concerted at 40 °C using an evaporator. The solution was desiccated in a drying oven for 1 h at 40 °C. The left over was stored in airtight bottles at 4 °C for further usage. Five distinct extracting procedures were used to test the extraction of bioactive substances. These procedures included supercritical fluid extraction (SCFE), liquid–liquid micro-extraction (LLME), microwave-assisted extraction (MAE), cold extraction (CE) and serial exhaustive extraction (SEE). The SCFE was carried out in a 15 ml capacity beaker with a sample (2.5 g). The temperature gradient in static was 45 °C for 25 min accompanied by the methylation process. The next extraction method used was microwave extraction. The content of both herbal materials was grinded using a mortar pestle and the samples were placed in a petri dish and heated completely in the oven. The samples were immersed in icy water once they had healed completely to minimize the heat. The goal of serial exhaustive extraction (SEE) was to increase the polarity spectrum of the solvent to ensure that the right amount of bioactive chemicals was extracted. The liquid- liquid micro-extraction method was used in which the solvent extract of both herbs (12–100) were incorporated into a vortex machine for achieving the interaction between the water and solvent and an equivalent concentration of both solvents was introduced into a capillary firmly connected to HPLC-DAD with tandem mass spectrometer (MS/MS) (Chhikara et al., 2019).

### 2.4 Producer of HPLC-DAD and approaches of column phase structure for the separation of active ingredients

HPLC apparatus is connected to a detector, as in a (DAD) which operates at a wavelength of 190–800 nm and is commonly used in MS/MS. A photodiode array detector and a binary pump is included in the HPLC system (MA, Milford, USA). A water process analytical Columns and an auto-injector are connected to system. The extract trial (5 ml) was steamed until completely dried then the leftovers were incorporated in a DMSO solution (2 ml).The chromatographic isolation was accomplished out on a waters X Bridge C18 column. The solvent A, water and solvent B methanol was utilized as mobile phase, which included 1.50% tetrahydrofuran as well as 0.25% orthophosphoric acid. The following were the gradient requirements; 0–15 min 30–70% A, 15–30 min 70–100% A, 30–35 min 100% A, 35–36 min 100–30% A, and 36–50 min 30%. Before each test, the column was evenly balanced for ten minutes. The mobile phase was tested using ultrasonic waves, before the samples were operated. The DAD-MS/MS and MS/MS were used to analyze the spectrum properties of bioactive substances between 190 and 800 nm. The spectral value was observed by both sensors, with their top value indicated in the inflexion of each point throughout the analysis of samples. Time is a critical component for an accurate study of bio- active chemicals in HPLC testing. Various mobile phases (aqueous 0.1% v/v, acetonitrile methanol, 0.1% formic acid, and 0.1% aqueous ethylic acid v/v) were used to optimize the extraction of bioactive chemicals with the ideal moment for monitoring. The best mobility phase for isolating and collecting four different aspects was found to be 0.1 %methanol and formic acid. Elution mode utilized formic acid (0.1%) and methanol with dissociation, a liner program of 10–65% B 0–85 min, and basic concoction components being used as standard of the specified identifiers (Benzie and Strain, 1996).

### 2.5 Evaluation, precision, and calibration methods for novel bioactive compounds found in medicinal plants

The proposed methodology was assessed effectively by generating optimal conditions for HPLC analysis. The peak area and percentage of the associated working criteria solution was plotted to construct calibration and linearity curves. Moreover, the applied analytical procedure was evaluated in terms of accuracy, reproducibility, consistency, recovery as well as the limit of quantification (LOQ) and limit of detection (LOD). The curves calibration was premeditated in each peak region of four bioactive components and the standard compounds were contrasted to the performance of sample of plants. The variables introduced into an equation y = axe ± b, with × represent in contractions and y representing peak area. The R2 values were used to verify linearity. In curve calibrations, the accuracy was tested at three different concentrations level. The intra and inter days were measured after 5 days of each sample replications were performed in HPLC-DAD/MS/MS and thus reliability of techniques as well as RSD was determined (Chhikara et al., 2019, Mani and Kumar, 2014).

### 2.6 Novel compounds ' for drug potency and biological activity at the cell levels of bacterials

#### 2.6.1 Bacterial strains studies (Gram positive & negative bacteria)

The study used *Staphylococcus aureus* and *Bacillus cereus* bacteria (two-gram positive) and *Escherichia coli a*nd *Klebsiella pneumonia* bacteria (two-gram negative) to test the putative antimicrobial activities of novel bioactive substances for influencing these contagious bacterial types and their recognition characters were *Klebsiella nomine* B.96.1, *Bacillus cereus* KA 80800, *Staphylococcus aureus* B.965 and *Escherichia coli* O157.

### 2.7 Diverse antibacterial activity of compounds

#### 2.7.1 Antibacterial strains' minimum inhibitory concentration (MIC)

The lowest bacterial contents measured and registered to both plants' novel compounds were measured It was the lowest content where an antibiotic or antibacterial chemical might kill a microbe. Using the agar test method, the MBC was calculated by placing the sample and bacillus on plate of agar. The aliquots (5 μl) to were placed to TSA plates and incubated for 24 hours, while the controls were loaded with DMSO solution in proportions equivalent to the maximum amount contained in a solution in which the correct study was done by the analysis of 3 replicates using the procedure described in (Gohari et al., 2011, Taylor et al., 1983, Owuama, 2017).

### 2.8 Combined fractional inhibitory concentration index (CFICI)

By using the formula FIC index = FICA + FICB, where FICA = (MICA in combination/MICA alone) and FICB = (MICB in combination/MICB alone), CFICI test was performed on solution obtained from plants (Contreras et al., 2015). The trails had been replicated at least 2 times with a copy for each sample to be run and analyzed separately.

### 2.9 Scanning the processing of a sample electron microscopy (SEM)

Following the final depletion of ethanol–water from herbal extracts were obatined then it was added to 50% ethanol for 30 min. The Hexamethyldisilazane/ethanol was substituted. The sample was deposited in a tiny droplet (0.05 ml) on perfectly polished carbon discs and held exposed in the hot spot to dry overnight. Carbon tape was used to secure the carbon discs on both sides of aluminum stubs. Experimental samples were administered for 45 min of ruthenium tetroxide treatments (Taylor et al., 1983). The sample patterns were tested by the Zeiss Ultra plus Field Emission Gun Scanning Electron Microscope (FEG-SEM).

### 2.10 Electron microscopy (TEM) sample processing and producer of bacterial strains

After the third stage of 100% exhaustion, for 2 h, ethanol was substituted with propylene. Epon epoxy resin was used to replace propylene oxide and it was applied for 5 hours. The micro-centrifuge tubes were opened, and the pellets were removed, inserted into molds and required 48-hour of polymerization. The segments were then slashed with a diamond knife on the Reichert Ultracut E ultra-microtome and taken from the copper network. The matrix was tarnished with uranyl acetate (2%) for 2 min before being stained with Reynold's lead citrate for 2 min. In the discipline of electron microscope the segments were seen and shot with a Philips EM 10 transmission microscope.

### 2.11 Serval antioxidants process of various herbal extracts

#### 2.11.1 Assay technique for ferric ion reducing antioxidant potential (FRAP)

The revised approach of (Ahmed et al., 2019) was used to evaluate the FRAP of two plants. The stock solutions, assembled with 300 mM acetate buffer (CH3COONa 3.1 ml and CH3COOH 16 ml), pH 3.6 and 2,4, 6-tripyridyl-s-triazine (TPTZ, 10 mM2) solution in HCl (40 mM) and FeCl3 (20 mM) were used. The new mixture was made by combining 25 ml acetate buffer, 2.5 ml TPTZ and 2.5 ml FeCl3 solution, then calculating the average activity of samples.

#### 2.11.2 Assay technique for ferric reducing antioxidant power (ABTS)

The procedure recognized inhibition of the production of the ABTS radical’s cation through some slight modification of 1 ml of ABST dye which was dissolved in acetate solution and pH were adjusted to pH 4.0 and then prepared with 1 ml potassium persulfate solution. The ABTS dye of 1 mg was used and prepared in 100 ml of a methanol solution having high water solubility and chemical stability of radicals during measurement process (Miller, 1996). The absorbance of ABST was recorded at 734 nm and the stability of reaction maintained by 1 mg of KCl salt was used in this activity.

### 2.12 Drugs designed based on compartment and non-compartment modelling of pharmacokinetics studies of compounds

Pharmacokinetic compartment modelling was used on these novel substances, which had been derived from *A. vasica* and *C. procera*. The half-life was determined by the regions under plasma concentration, while the peak plasma concentration as well as timing acquired immediately from the dataset obtained in this investigation. Medicinal phyto-constituents were incorporated to the compartment model and an automatic calculating software tool was coupled with win-Nonlin ® Software to these bioactive chemicals. The region under plasma levels was estimated AU Clast- = Clast/Kel for modern drug capability and values of these components were assessed as described in (Mani and Kumar, 2014), comparable to the solubility and other essential properties were determined as procedure described of (Vij and Prashar, 2015, Cakir et al., 2004).

### 2.13 Non-Compartment to a One-compartment model for new antibiotic process

Selected compounds were administered using a one-compartment oral dosage model, extract the one-compartment model parameters, the non-compartment model Parameters are necessary. The equations for non-compartment and one-compartment variables are given below respectively.

(1) w = (AUC, T max, T1/2)
(2) β = (ka, ke, V).
(3) β = (ke, V)
(4) CL po = V × Ke

If only oral clearance is required, CL po should be used instead of AUC. When it comes to IV dosage, however, just w = (AUC, T1/2) is required to claim the single dozes changes in (3).

Plasma concertation either orally or via an injection of selected compounds The natural antibiotic compounds by being taken into cells and either bacterial changes and it formed a tragedy to cells as expressed complete changes used in the formation of ATP in cells. The pharmacokinetic description of antibiotic compounds are shown in below equation and represented mathematically by equations (1), (2), (3), (4).

(1) dSdt=rS,end+rS,ex-rS,elim

(2) rS,end=f(t,S,I,G,E,F,A,KS,end)

(3) rS,ex=g(t,MS)

(4) rS,elim=h(t,S,I,E,KS,elim)

### 2.14 Low electrical cell membrane permeability of selected bacteria changes in cells

The presence of bacterial cells is reflected in the flow of electrical energy and is determined in the manner (Ahmed et al., 2021). After planting, the bacteria were purified with 5% glucose till their electrical power reached a level comparable to 5% glucose. There were isotonic bacteria in two separate MIC and 2 × MIC areas, also introduced to glucose (5%) as well as a solution of isotonic bacteria correspondingly. Following the complete mixing, the samples were placed for 1–2 h at 37 °C. Infections caused by viruses was recorded by a formula previously given by (Li et al., 2005). The effective entrance of bacteria (cm / s) was calculated using concentration data as seen below (4) where A is the filter input area (0.3 cm^2^), VD volume supplier (0.3 ml), VA volume receiver/receiver well (0.2 ml), CA (t) concentrations well-received during t, and incubation time (18000 s). The following expression is used to determine Ceq:

The four equation with parts of linked to above equations.

The other equation is suitable for CD is divided its written in below.

(5) CD(t)

At time t, the concentration in the donor well is CD (t), as well as the other values are the same as before.

### 2.15 The integrity of the cell membrane activity and changes of bacterial cells by novel antibiotic

The integrity of all bacterial strain types was assessed by detecting intracellular material leakage as described previously (Ahmed et al., 2021)with minor alterations. S. aureus cells from a 100 ml operational culture were examined and centrifuged for 10 min at 6000 rpm. After three washes, the samples were returned to a phosphate buffer solution with a pH of 7.4 and a concentration of 0.1 M. A hundred milliliters of cell suspension were deposited in three separate places (0, MIC, and 2 MIC) at 37 °C with 4 h pressure. Following that samples (20 ml) were taken and 10,000g was extracted in 5 min. Also, concentration of sugar reduction in the supernatant was indicated by the DNS color reaction and also in comparison to the glucose calibration curve the body index showed 575 nm absorption. Moreover, to detect the accumulation of highly concentrated UV components such a nucleic acid and protein and UV absorbance at 280 nm and 260 nm was measured using a supernatant (3 ml). The change was done to absorb a suspension in the same PBS with same sample density.

### 2.16 Fluorescence of membrane strength of bacteria changes with antibiotic compounds

The membrane strength was measured as previously reported for moderate modifications (Khan et al., 2012). At 37 °C, the bacterial samples were cultured for 10 h in nutritional broth. The suspension was introduced to MIC and 2MIC for ME, correspondingly, whereas the control culture was kept unaffected. Afterward, the suspension was kept at 37 °C for 2 h respectively. Prior to testing, DiBAC4 (3), a sensitive fluorescent membrane processor, being introduced to the cell solution at a final concentration of 0.5 g / ml. Fluorescence strength was measured after 5 min using a customized fluorescence microscope (Leica DMi8, Wetzlar, Germany).

### 2.17 New collagen binding assay methods

A slightly modified technique described by Aligiannis et al. (2003) was used to examine the bacteria's ability to bind to placental collagen. Specifically, 250 ml of 50 μg/mL sheep and human placental collagen solutions were assaulted individually on 8-well chamber slides using the Permanox®, Nunc® Lab-Tek® method was adopted.

### 2.18 Compound cytotoxicity testing and putative activities

The samples were investigated with the help of an LDH kit. The emission of lactatdehydrogenase was determined using the calorimetric method described by Muzitano et al. (2006) using the extract of plant samples (2 ml). The concentrations varied and the sample was kept for 24 h. 1% X-100 Trition and DMSO solution was mixed in a comparable ratio to make the lysates. The procedure was further dispersed, and control samples were compared.

### 2.19 Lactate dehydrogenase (LD) activity assessment of novel bioactive chemicals for antibacterial medicines in vitro

Due to membrane breakdown the cytosolic enzyme LDH was secreted and the membrane integrity was then evaluated by quantifying the amount of LDH in pure culture. LDH release in relevant cells was measured using the cytotoxic 96 X assay. 50 μl of revived reactant were mixed with same amount of cell culture media then cultured in dark for 30 min. At 490 nm, colorimetric compound was detected spectrophotometry (Perkin-Elmer, VICTOR3).

### 2.20 Scanning the processing of a sample electron microscopy (SEM) repeated

After final depletion of ethanol–water, hexamethyldisilazane (HMDS) was applied to ethanol at a 50% concentration for 30 min. HMDS was applied twice after each hour to substitute hexamethyldisilazane/ethanol. The sample was deposited in a tiny droplet (0.05 ml) on perfectly polished carbon discs and held exposed in the hot spot for drying. Carbon tape was used to adhere these carbon discs to both sides of aluminum stubs. For 45 min, experimental samples were subjected to ruthenium tetroxide (RuO4) (Taylor et al., 1983). The samples were tested with the Zeiss Ultra Plus Field Emission Gun Scanning Electron Microscope (FEG-SEM).

### 2.21 Electron microscopy (TEM) sample processing for novel antibiotic compounds changes

After the third stage of 100% exhaustion ethanol was substituted with propylene for 2 h. Epon of epoxy resin (TAAB 812) was used to replace propylene oxide and was applied for 5 h. The pellets were taken out of the centrifuge tube, placed in molds and then polymerized for 2 days. The sections were then slashed with a diamond knife on the Reichert Ultracut E ultra microtome and removed from the copper grids. The grid was stained with uranyl acetate (2%) for 2 min before being stained with Reynold's lead citrate for 2 min. In the discipline of Electron Microscopy, the sections were seen and shot with a Philips EM 10 transmission microscope.

### 2.22 Statistical analysis

Knauer's Chrom gate v 3.31 software was used to statistically evaluate the collected data. Meanwhile graph pad prism 5 used non-linear analysis for each sample’s concentration–response curve to establish the minimum bactericidal concentration (MBC). The standards digital library was used to double-check the chemicals. Additionally; the fingerprinting of novel chemicals was assessed by Sciex City Foster USA. Modern software from Multi-Quant 2.1 was used to evaluate the results for monitoring, data acquisition, peak measurements and verification. Win Non-Lin® version 6 software was used to create peak regions, standards curve and descriptive analysis.

## 3 Results

The accuracy and efficiency of extracts of *A. vasica* and *C. procera* in few solvents suchas ascorbic acid, chloroform, ethyl acetate, methanol, and hexane, as well as to validate the involving biological methodologies of non-compartment and compartment modelling of pharmacokinetics, several isolation methods were applied to S-allylcystein, Myristyl diglucoside, D- Triglucopyranoside, and B-Sitosteryl linoleate.

### 3.1 HPLC-DAD investigation of antibiotic drugs' effectiveness based on solvents as a drug method

**Fig. 1:**
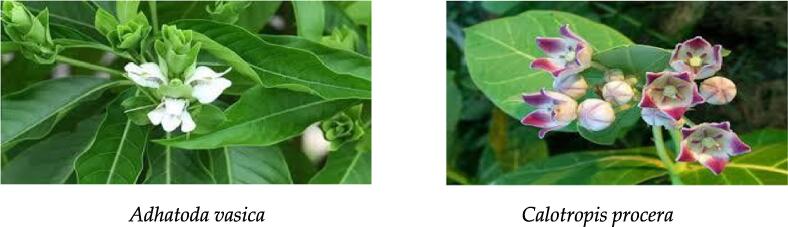
The medicinal plants as novel compounds.

**Fig. 2:**
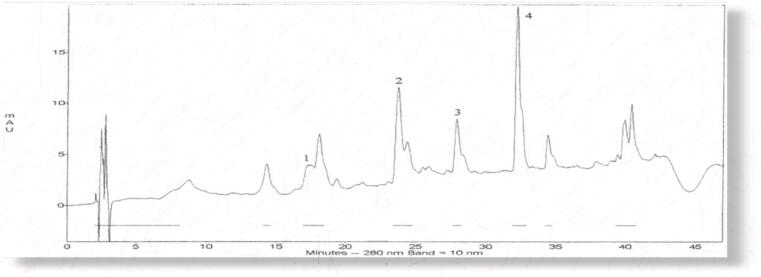
HPLC-DAD/MS/ chromatograph of compounds peaks indicated as follows: (1) B-Sitosteryl linoleate (2) Myristyl diglucoside (3) 2 D-Triglucopyranoside (4) S-allylcysteine acid A (Adhatoda vasica) and B (Calotropis procera).

**Fig. 3:**
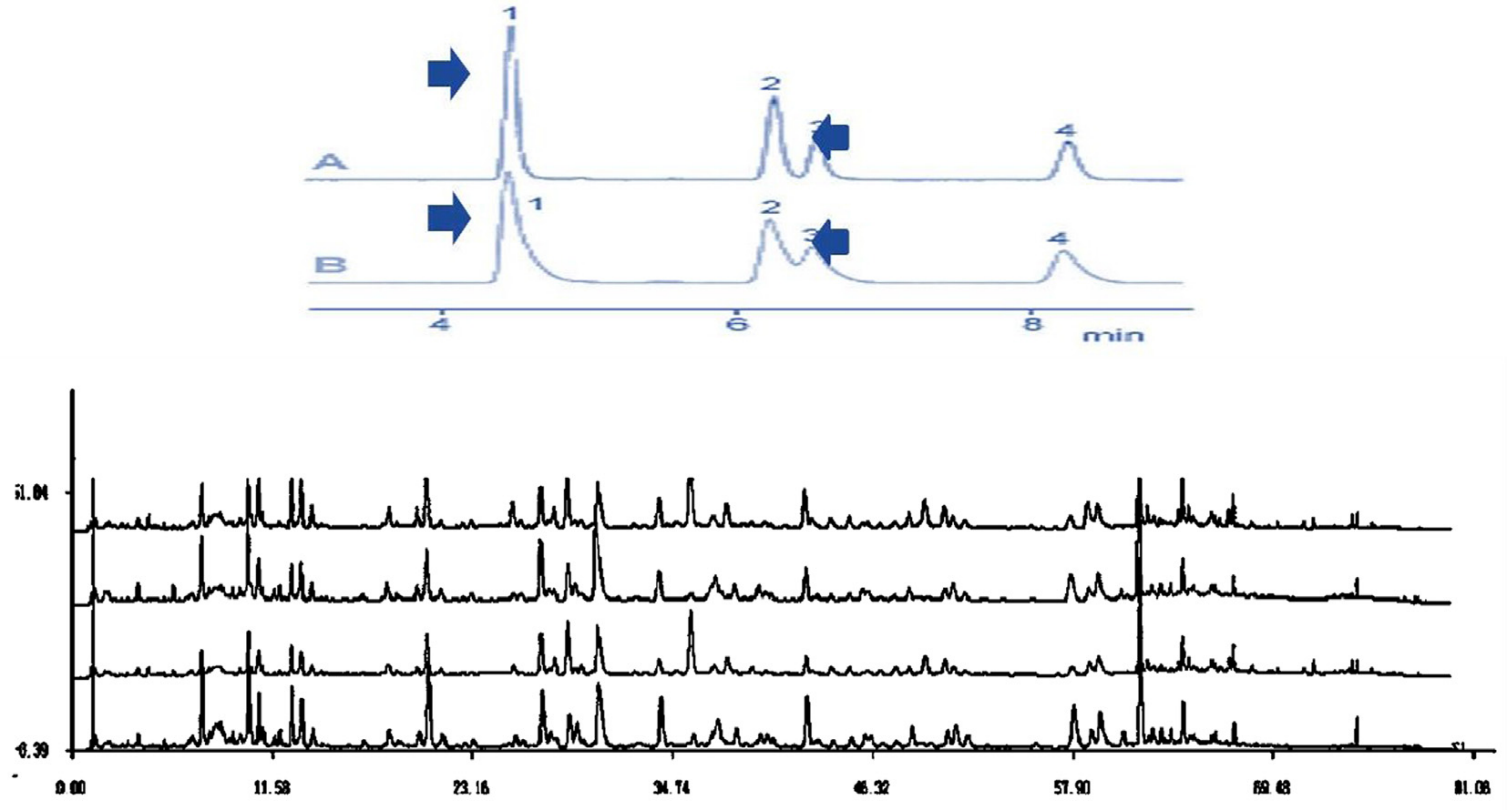
Chemo-fingerprinting chromatograms confirmation of with numerical A (Adhatoda vasica) and B (Calotropis procera) as follows follows (1) B-Sitosteryl linoleate (2) Myristyl diglucoside (3) 2 D-Triglucopyranoside (4) S-allylcysteineacid.

Using HPLC analytical method, the quantification of novel bioactive compounds (Myristyl diglucoside D-Triglucopyranoside, S-allylcysteine acid, B-Sitosteryl linoleate) were identified in *A. vasica* and *C. procera* samples and investigated in six different solvents. D-Triglucopyranoside (13.81 ± 0.48%), Myristyl diglucoside (11.81 ± 0.41%), B- Sitosteryl linoleate (12.81 ± 0.48%), and s-allylcysteine acids (14.81 ± 0.31%) were detected in large concentrations in methanol extract. In contrast, ascorbic acid solvent extracts had lower percentages. With outputs of (13.81 ± 0.42%) and (13.80 ± 0.41%),*C. procera* was also discovered to be the best source of Myristyl diglucoside and Sitostery linoleate chemicals shown in Table 1. *A. vasica* and *C. procera* were shown in Fig. 1. The chromatogram of both plants' methanolic extracts was shown in Fig. 2 and Fig. 3 where four peaks were used with numeric values. Likewise, the highly sophisticated software utilized in this investigation displayed the needed fingerprinting of four compounds in Fig. 3 (see Table 1).

**Table 1:** Solvent based efficiency of B-Sitosteryl linoleate, Myristyl diglucoside, D-Triglucopyranoside, Sallylcysteine acid from Adhatoda vasica and Calotropis procera as novel compounds.

		Novel antibiotic compounds	(%)		
Herbal leave extracts	Several Extraction solvents	linoleate	diglucoside	Triglucopyranoside	Sitostery linoleate
Adhatoda vasica	Methanol	12.81 ± 0.48	11.81 ± 0.41	13.81 ± 0.48	14.81 ± 0.31
	Ethyl acetate	8.11 ± 0.17	7.11 ± 0.16	9.11 ± 0.11	7.11 ± 0.14
	Chloroform	9.72 ± 0.06	8.72 ± 0.05	8.72 ± 0.04	7.71 ± 0.03
	Hexane	7.1 ± 0.02	6.92 ± 0.01	6.2 ± 0.02	6.91 ± 0.01
	Aqueous	6.91 ± 0.03	5.90 ± 0.02	5.81 ± 0.03	5.90 ± 0.02
	Ascorbic acid	5.91 ± 0.04	4.99 ± 0.02	3.92 ± 0.04	2.99 ± 0.02
Calotropis procera	Methanol	13.81 ± 0.46	13.81 ± 0.41	12.81 ± 0.46	11.81 ± 0.41
	Ethyl acetate	9.11 ± 0.15	8.11 ± 0.16	9.11 ± 0.15	8.11 ± 0.16
	Chloroform	8.71 ± 0.05	7.72 ± 0.05	8.71 ± 0.05	7.72 ± 0.05
	Hexane	6.1 ± 0.03	6.96 ± 0.01	6.1 ± 0.03	6.90 ± 0.01
	Aqueous	5.91 ± 0.02	5.96 ± 0.03	5.92 ± 0.02	5.91 ± 0.03
	Ascorbic acid	4.91 ± 0.03	4.92 ± 0.02	13.81 ± 0.48	14.81 ± 0.31

Mean of three determinations with ± SD of medical plants.

### 3.2 Efficient methods for extracting antibiotic chemicals for the drug manufacturing process

The five innovative extracting techniques have been employed for achieving productivity and viability of substances. The precise quantification method enlisted in Table 2. Four compounds such as Myristyl diglucoside, S-allylcysteine acid, D-Triglucopyranoside and B-Sitosteryl linoleate had higher yield and precision. With the computation of B-Sitosteryl linoleate, Myristyl diglucoside, compounds in *A. vasica* given in Table 3. SCFE (12.11 ± 0.17) was the most efficacious method. Table 2 shows similar patterns in *C. procera*. SCFE showed the highest percentages (Table 2) of four compounds in both medicinal plants. Separation speed, chiral separation, and lower mobile phase viscosity are all significant values of SFC. The second standard strategy was LLME of all four components computation and quantification procedure in comparison to another three ways of exactions in both herbal plants. The liquid extraction method was used for obtaining higher and purer extracts for a purified and higher isolation procedure with a yield potential of chemicals. MAE has a myriad of benefits, notably raising extract yield, limiting thermal degradation, and precise heating of vegetal material.

**Table 2:** Methods extraction of B-Sitosteryl linoleate and Myristyl diglucoside D-Triglucopyranoside allylcysteine acid from Adhatoda vasica and Calotropis procera.

Herbal Extracts	Extraction methods	B-Sitosteryl linoleate,	Myristyl diglucoside	D-Triglucopyranoside	S-allylcysteine acid
*Adhatoda vasica*	Cold extraction (CE)	11.81 ± 0.48	11.81 ± 0.41	12.81 ± 0.48	11.81 ± 0.41
	Super critical Fluid extraction (SCFE)	12.11 ± 0.17	12.11 ± 0.11	18.11 ± 0.17	17.11 ± 0.16
	Microwave assisted extraction (MAE)	9.72 ± 0.06	8.72 ± 0.01	9.72 ± 0.06	8.72 ± 0.05
	Exhaustive extraction (EE	7.1 ± 0.02	6.92 ± 0.01	7.1 ± 0.02	6.92 ± 0.01
	Liquid-liquid micro- extraction (LLME)	6.91 ± 0.03	5.90 ± 0.02	6.91 ± 0.03	5.90 ± 0.02
*Calotropis procera*	Cold extraction (CE)	13.81 ± 0.46	13.81 ± 0.41	13.81 ± 0.46	13.81 ± 0.41
	Super critical Fluid extraction (SCFE)	9.11 ± 0.15	8.11 ± 0.16	9.11 ± 0.15	8.11 ± 0.16
	Microwave assisted extraction (MAE)	8.71 ± 0.05	7.72 ± 0.05	8.71 ± 0.05	7.72 ± 0.05
	Exhaustive extraction (EE	6.1 ± 0.03	6.96 ± 0.01	6.1 ± 0.03	6.96 ± 0.01
	Liquid-liquid micro- extraction (LLME)	5.91 ± 0.02	5.96 ± 0.03	15.91 ± 0.02	14.96 ± 0.03

Means of three determinations ± SD of species.

### 3.3 Specific parameter of HPLC-DAD/MS of four chemicals with the proper measurement process (LOD & LOQ)

Limit of Quantification (LOQ) and Limit of Detection (LOD) methods of 4 reactive substances were evaluated in three doses, as well as findings of each analyte's LOD and LOQ are reported in Table 3, Table 4. S-allyl cysteine acid had a greater LOD (1.3) and a lower LOQ (0.9) in the results. Furthermore, the outcomes suggest that the calibratio curves of 4 substances have appropriate ranges. Table 3, Table 4 illustrate the specificity o HPLC-DAD/MS/MS for every sample, examined through inter and intraday accuracy.The relative standardized deviations (RSD) of four active ingredients were (0.9% Intraday and (0.98%) intraday.

**Table 3:** Accuracy and validations of compounds data of chromatographic separation via HPLC-DAD methods.

Antibiotic Bioactive Compounds	tR (min) a	b (nm)b	LOD: detection limit c	LOQ: quantification limit d	Linear range (mol/l)	(r)a	Intraday (RSD%)b	Interday (CV%) c	Accuracy (%)
B-Sitosteryl linoleate,	4.11	280	0.8	1.2	0.19-9.25	0.967	0.6	1.12	101.1
Myristyl diglucoside	10.13	280	0.9	0.5	0.22-10.11	0.968	0.8	0.24	102.2
D-Triglucopyranoside	7.11	280	1.0	0.8	0.23-10.75	0.988	0.7	1.25	98.4
S-allylcysteine acid	15.14	280	1.3	0.9	0.21-17.51	0.962	0.9	0.98	94.3

General LOD = 1.22, General LOQ = 1.23 a tR: retention time, b: wavelength, c LOD: detection, limit, d LOQ: quantification limit, a correlation coefficient, b RSD: relative standard deviation, c CV: coefficient of variation.

**Table 4:** Stability Precision, Analysis of recovery of compounds by (HPLC- DAD) methods.

Antibiotic Bioactive Compounds	Original in mg	Detection (mg)	Addition (mg)	RSD ^a^ (%)	RPA	RRT	Recovery ^b^ (%)
B-Sitosteryl linoleate	4.12	4.101	1.12	0.121	4.12 ± 0.512	4.12 ± 0.512	100.1
Myristyl diglucoside	10.11	10.11	1.25	0.123	4.12 ± 0.512	4.11 ± 0.01	101.2
D-Triglucopyranoside	7.11	7.21	1.23	0.551	10.12 ± 0.513	10.13 ± 0.02	99.1
S-allylcysteine acid	15.10	15.11	0.12	0.121	7.12 ± 0.501	7.11 ± 0.01	98.1

The data was present as average of three trails. a RSD (%) = (SD of amount detected/mean of amount detected) × 100. b Recovery (%) = 100 × (amount detected – original amount)/addition retention area and relative retention time, SD is Presented as replicate of three trails.

### 3.4 Reliability of novel bioactive compounds using a chromatographic separation process using the HPLC techniques

#### 3.4.1 Biological activity of new antibiotic compounds responses for drug manufacture process, Agar Dis Diffusion method of Minimum bacterial concentrations (MBCs), minimum inhibition concentrations (MIC), combined fractional inhibitory concentration index (CFICI) of selected bacterial strains

**Fig. 4:**
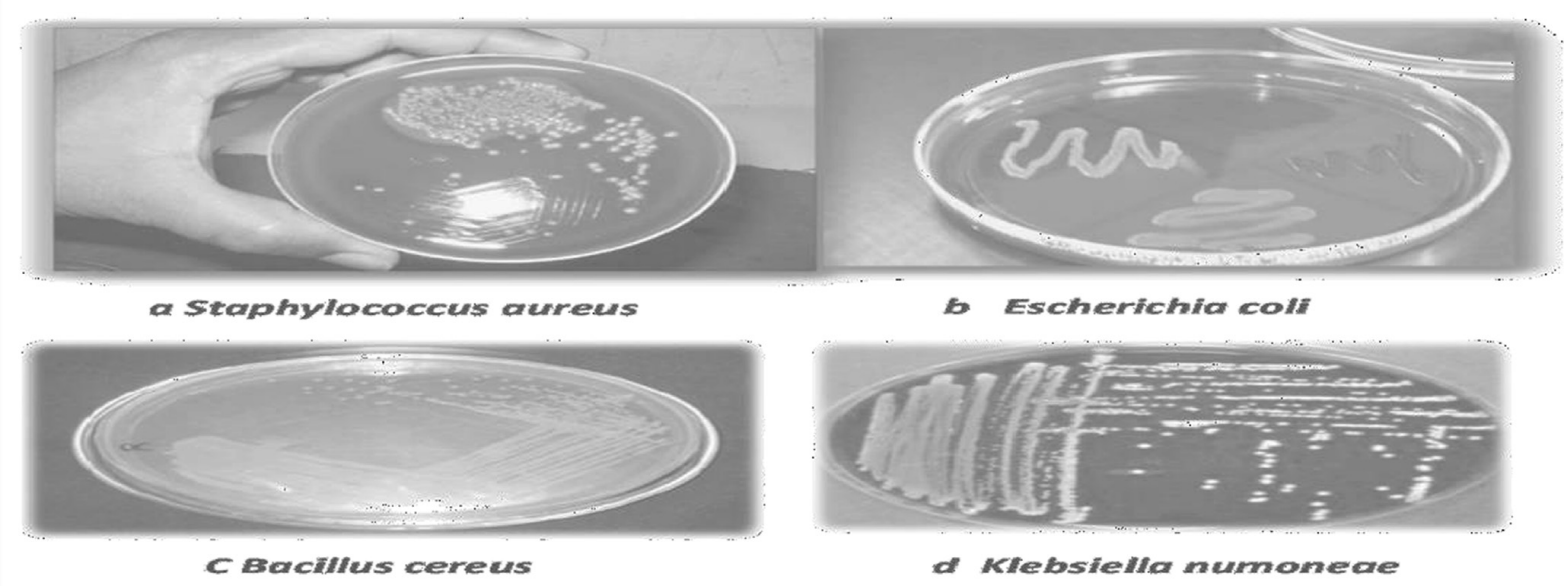
Agar diffusion method of MBC activity (a, b, c and d) from Adhatoda vasica Calotropis procera.

**Fig. 5:**
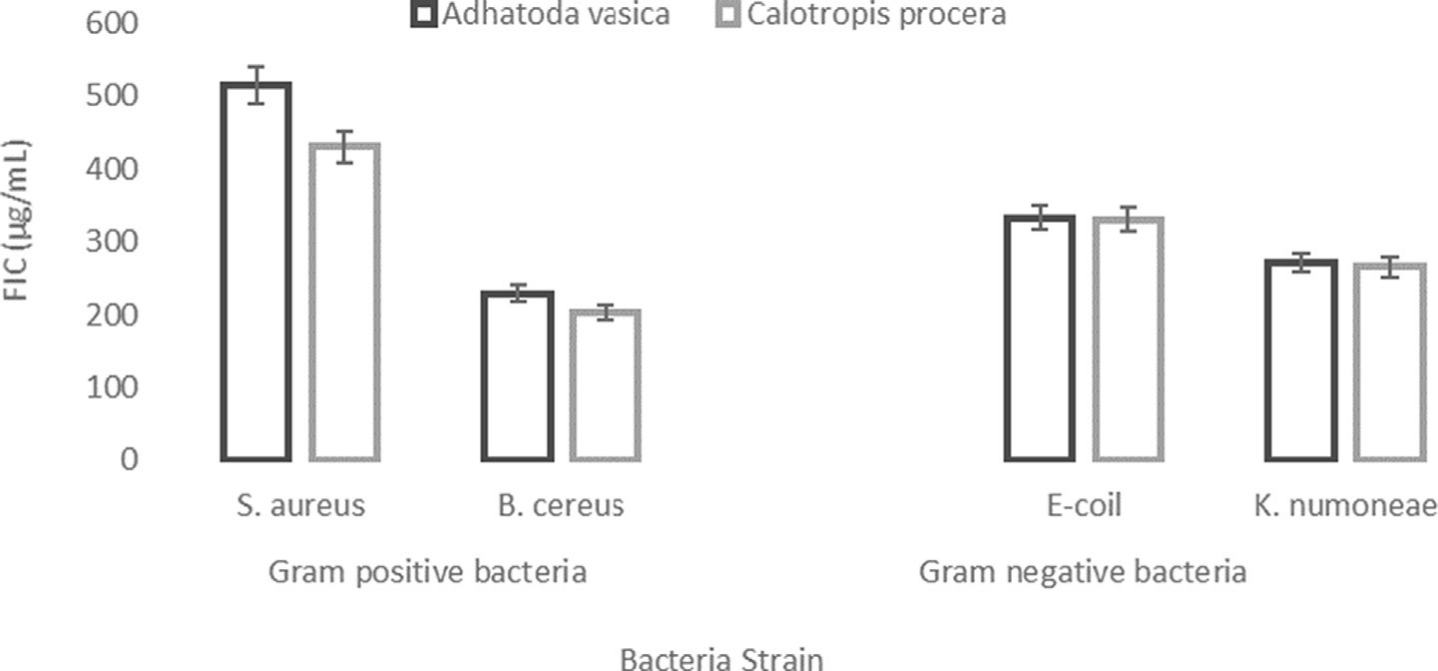
Fractional inhibitory concentration (FIC) for different bacterial strains Each vertical bar represents mean of three replicates ± S.E.

The agar dis diffusion test was used to analyze bacterium strains into four individual elements of each plants and methanol extract was used to purify them as given in Table 5. The MBC activity of *S. aureus* was 520 g/ml and B. cereus was 230 g/ml as shown in Fig. 3. The MBC for E-coil was 335 g/ml and *K. numoneae* was 272 g/ml, from *A. vasica* isolated leaves solutions. The MBC values against *B. cereus*, *S. aureus*, *K. numoneae and E-coil* were203 g/ml, 432 g/ml, 266 g/ml and 331 g/ml, in *C. procera* leaf extracts, respectively. Fig. 4 also shows the MCB ranges with their variants. These results indicated that the extracted 424 maximal CFICI evaluation towards S. aureus (positive bacteria) was (515 μg/m). In comparison to the active concentration against B. cereus (203 g/ml), the *C. procera* leaves solution showed improved effectiveness against S. aureus with 432 g/ml. *E-coil* and *K.numoneae* have CFICI values of 333 and 265 g/ml against a negative bacterial strain. Fig. 5 depicts the results. Chemicals had better antibacterial activity. Based on the combined fractional inhibitory concentration index (CFICI) of four compounds, Table 5 demonstrates that methanol extracts from plants are particularly efficient against bacteria. In *A. vasica* (leaves extract).

**Table 5:** Various methods of MBCs) minimum bacterial concentrations MIC minimum inhibition concentrations (CFICI) Combined fractional inhibitory concentration index of herbal extracts.

Bacterial Strain	Various methods of antibacterial activity					
	(MBCs) (μg/ml)		MIC(μg/ml)		(CFICI) (μg/ml)	
	*Adhatoda vasica*	*Calotropis procera*	*Adhatoda vasica*	*Calotropis procera*	*Adhatoda vasica*	*Calotropis procera*
Gram positive bacteria						
*Staphylococcus aureus*	520	432	521	433	515	432
*Bacillus cereus*	230	203	230	206	229	203
Gram negative bacteria						
*Escherichia coli*	335	331	338	333	333	330
*Klebsiella numoneae*	272	266	272	267	271	265
*Salmonella enterica*	150	100	150	104	333	330

Minimum bacterial concentrations (MBCs) (μg/ml) MIC (μg/ml), minimum inhibitio concentrations (CFICI) (μg/ml), combined fractional inhibitory concentration index Mean of three determinations ± SD of species.

### 3.5 Novel agents or antibiotics on changes under morphological and altercations changes of bacterial strains SEM

**Fig. 6:**
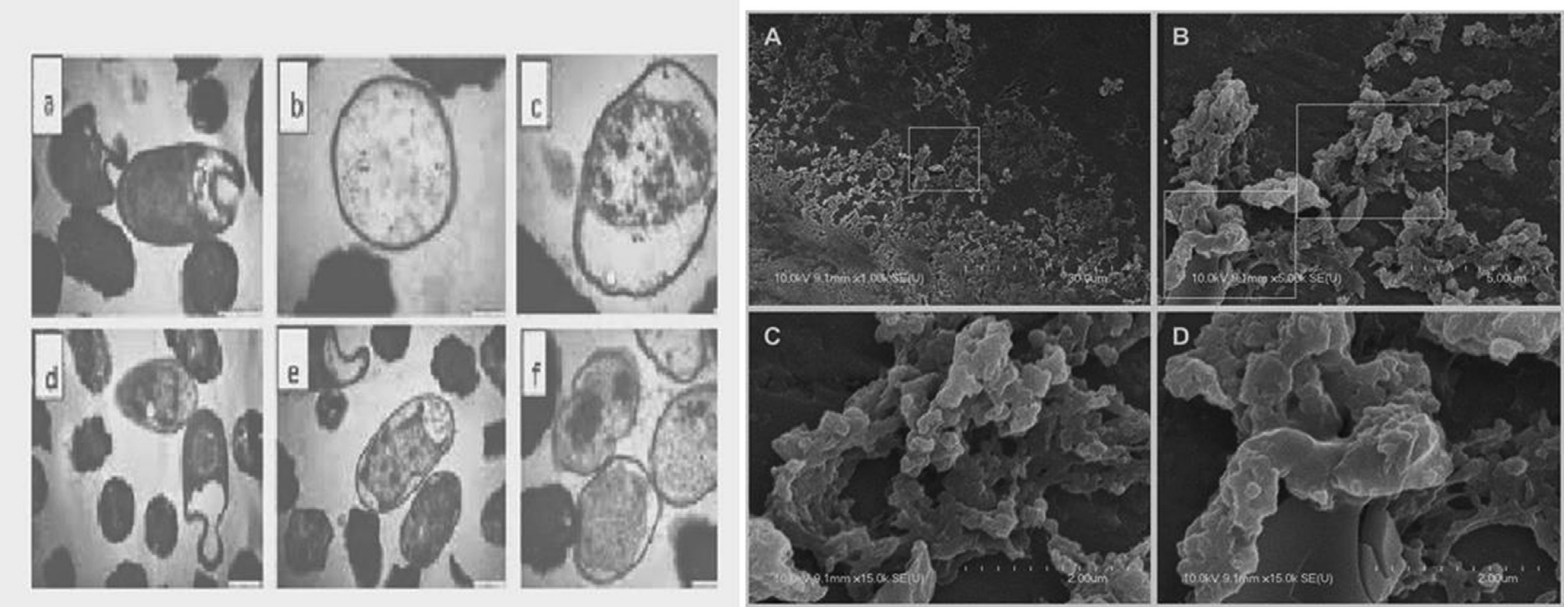
Altercation changes of bacterial responses of various antibiotic compounds, B-Sitostery Myristyld D-Triglucopyranoside S-allylcysteine *A. vasica* and in first figure are control reaming b-f five bacterial strains of *C. procera* by transmission electron microscopy (TEM).

**Fig. 7:**
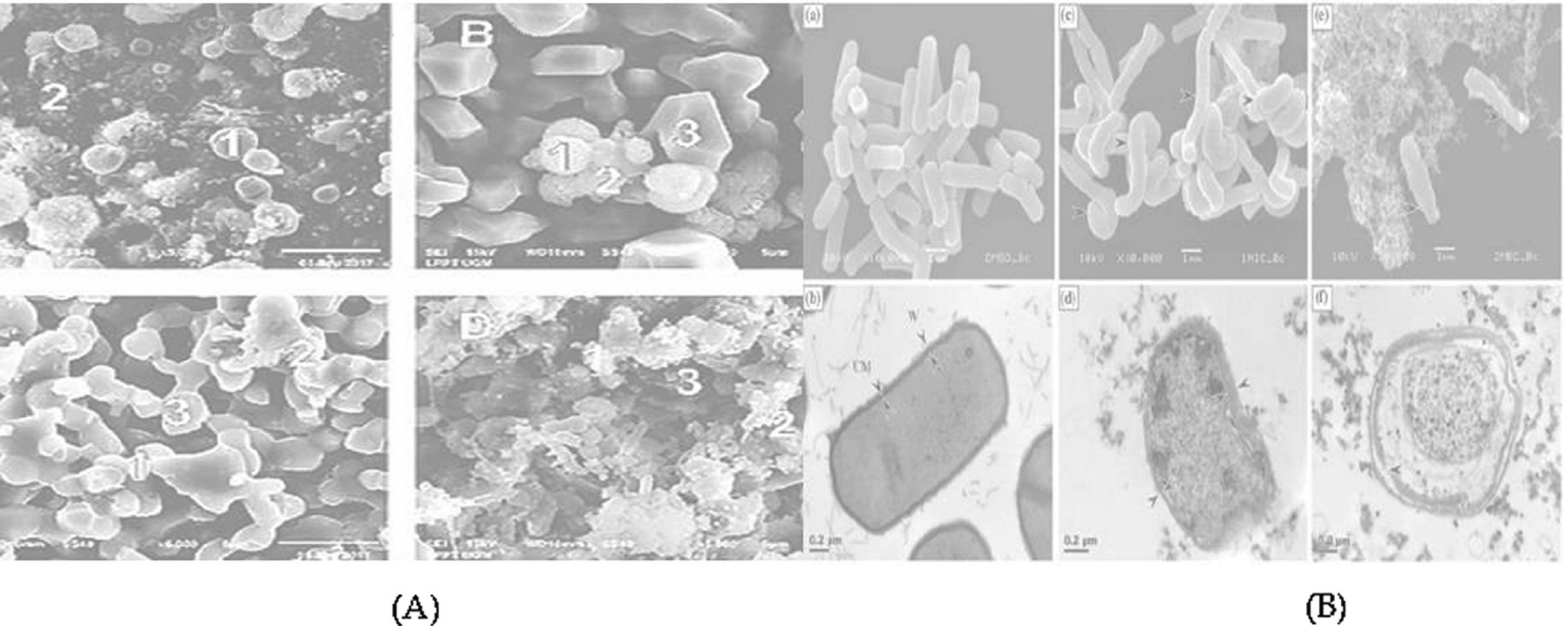
a and b. Scanning and transmission electron microscope easements of infectious index changes in (a-d) of fours compounds and five bacterial cells one as control are used by liquid extracts of *A. vasica* and *C. procera* of transmission electron microscopy (TEM).

In TEM Figs. 6 and 7a, b, the impact of methanolic extracts of each plant on cell morphologyand surface, cell wall, cytoplasmic membrane, cell division and cell viability of bacterial species were examined. The cell surface is rod-shaped and had smooth structural changes under microscopic analysis of antibacterial properties. The structure of cells was observed after application of liquid extracts on bacterial variants, *S. aureus* and *B. cereus* as well as *E. coil* and *K. numoneae with* rod-shaped, the length of cell was reduced with implying the cell shrinkage. The micrographs revealed that the cells were coarse and profuse cell membrane in the cytoplasm. According to a review of the literature, fila mentation occurs when rod-shaped bacteria create peptidoglycan for their lateral wall but not for the septal wall during growth, resulting in infections.

### 3.6 Antioxidants activity of ferric ion reducing antioxidant potential (FRAP) assay, totalantioxidant capacity (TAC) antioxidant power (ABTS) assay activity in medical plants

The antioxidant capacity of an extract made by the leaves of *A. vasica* and *C. procera* plants was measured in different solvents using the FRAP assay, with the outcomes presented in Table 6. Methanol extraction of *Adhatoda vasica* yielded the highest ferric ionlowering antioxidant potential. 12.81 ± 0.40 mg TE100 g^−1^, while ascorbic acid (2.910.03TE100 g^−1^) had lower levels. *C. procera* was determined to be the richest provider of active antioxidants, as evidenced by ferric ion reducing antioxidant potential (13.81 ± 0.41) mgTE100 g^−1^, while Ascorbic acid (2.91 ± 0.01) had lower FRAP values. The declining order of FRAP for various solvents is, methanol > Ethyl acetate > Chloroform > Hexane > Aqueous > Ascorbic acid. The total antioxidant activity of *A. vasica* methanol extraction was determined to be 12.41 ± 0.41%, while ascorbic acid (2.31 ± 0.1%) had lower levels. Likewise, all solvents had the same efficiency as the FRAP assay techniques. The methanol extraction of *Adhatoda vasica* yielded the highest (ABTS) concentration (11.81 ± 0.40 mg 488 TE100 g^−1^), meanwhile ascorbic acid yielded the lowest (2.91 ± 0.01 TE100 g^−1^).

**Table 6:** Antioxidants Activity FRAP, TAC, and ABTS assay of *A. vasica* & *C. procera* by different extractions solvents.

Preparation of solvents	Several of Antioxdants					
	Antioxidants Activity FRAP assay		Total antioxdants capacity (TAC)		Ferric reducing antioxidant power (ABTS) assay	
	*Adhatoda vasica* (mg TE100g^−1^)	*Calotropis procera* (mg TE100g^−1^)	*Adhatoda vasica* (mg %	*Calotropis procera*	*Adhatoda vasica* (mg TE100g^−1^)	*Calotropis procera* (mg TE100g^−1^)
Methanol	12.81 ± 0.40	13.81 ± 0.41	11.81 ± 0.41	13.81 ± 0.41	13.81 ± 0.41	13.81 ± 0.41
Ethyl acetate	12.11 ± 0.11	11.11 ± 0.12	12.11 ± 0.12	12.11 ± 0.12	11.11 ± 0.12	11.11 ± 0.12
Chloroform	11.71 ± 0.03	10.71 ± 0.02	11.71 ± 0.02	9.71 ± 0.02	10.71 ± 0.02	10.71 ± 0.02
Hexane	10.92 ± 0.01	9.92 ± 0.01	102 ± 0.01	8.92 ± 0.01	9.92 ± 0.01	9.92 ± 0.01
Aqueous	3.91 ± 0.02	1.91 ± 0.03	3.91 ± 0.03	2.91 ± 0.03	1.91 ± 0.03	1.91 ± 0.03
Ascorbic acid	2.91 ± 0.03	2.91 ± 0.01	2.91 ± 0.01	1.91 ± 0.01	2.91 ± 0.01	2.91 ± 0.01

Mean of three determinations of ± SD of herbal.

### 3.7 Bio-kinetics potential of permeability of cell membrane assay, the integrity of the cell

#### 3.7.1 Membrane and binding and infectious index

Results from the six essential important parameters of bacterial strains were measured, the Permeability of cell membrane essay of bacterial strains was higher in *Klebsiella numoneae* (14.92 ± 0.01) as followed by Staphylococcus aureus and lower in Salmonella enterica in leave extracts of *A. vasica*. Integrity of cell membrane of bacteria (11.81 ± 0.41) was higher in Staphylococcus aureus and less in (2.91 ± 0.03) in Salmonella enterica shown in Table 7. The Membrane potential and Bacteria binding activity were higher in all 500 strains of bacteria, the infectious index of all bacterial strains was in the range of 2–3%. The similar in *C. procera* leave extract showed the cell membrane permeability of bacteria range was 3–12, while all parameters of integrity of cell membrane of bacteria was higher in all selected strains. The infectious index was less in both leave extracts which ranges in between 1 and 3% however the leaves extracts of both plants have a potential of permeant stability and flexibility and member permeabilizing properties as shown by the increase in the percentage of dye leaking from the membrane with time.

**Table 7:** Response of liquid extracts on altercation of permeability of cell membrane of bacteria, integrity of cell membrane of bacteria at cell level.

Bacterial strains	Adhatoda vasica Extracts Bacterial changes in cells					Calotropis procera Extracts Bacterial changes				
	Permeability of cell membrane of bacteria	Integrity of cell membrane of bacteria	Membrane potential	Bacteria binding activity	Infectious index	Permeability of cell membrane of bacteria	Integrity of cell membrane of bacteria	Membrane potential	Bacteria binding binding	Infectious index
*Staphylococcus aureus*	13.81 ± 0.40	11 0.81 ± 0.41	10.81 ± 0.40	6.81 ± 0.40	3.81 ± 0.40	12.81 ± 0.40	11 0.81 ± 0.41	10.81 ± 0.40	6.81 ± 0.40	2.81 ± 0.40
*Bacillus cereus*	11.11 ± 0.11	10.11 ± 0.12	13.11 ± 0.11	4.11 ± 0.11	4.11 ± 0.11	10.11 ± 0.11	10.11 ± 0.12	13.11 ± 0.11	4.11 ± 0.11	311 ± 0.11
*Escherichia coli*	12.71 ± 0.03	11.71 ± 0.02	12.71 ± 0.03	3.71 ± 0.03	2.71 ± 0.03	11.71 ± 0.03	11.71 ± 0.02	12.71 ± 0.03	3.71 ± 0.03	1.71 ± 0.03
*Klebsiella numoneae*	14.92 ± 0.01	9.92 ± 0.01	11.92 ± 0.01	8.92 ± 0.01	4.92 ± 0.01	13.92 ± 0.01	9.92 ± 0.01	11.92 ± 0.01	8.92 ± 0.01	2.92 ± 0.01
*Salmonella enterica*	4.91 ± 0.02	2.91 ± 0.03	4.31 ± 0.02	2.91 ± 0.02	2.91 ± 0.02	3.91 ± 0.02	2.91 ± 0.03	4.31 ± 0.02	2.91 ± 0.02	2.91 ± 0.02

Membrane potential, binding activity and infectious of selected bacterial strains from *Adhatoda vasica and Calotropis procera* Mean of three determinations of ±SD of herbal plants compounds as used in medicines.

### 3.8 Drugs design and compartment modelling in pharmacokinetic study of substances for suitable medicines action

**Fig. 8:**
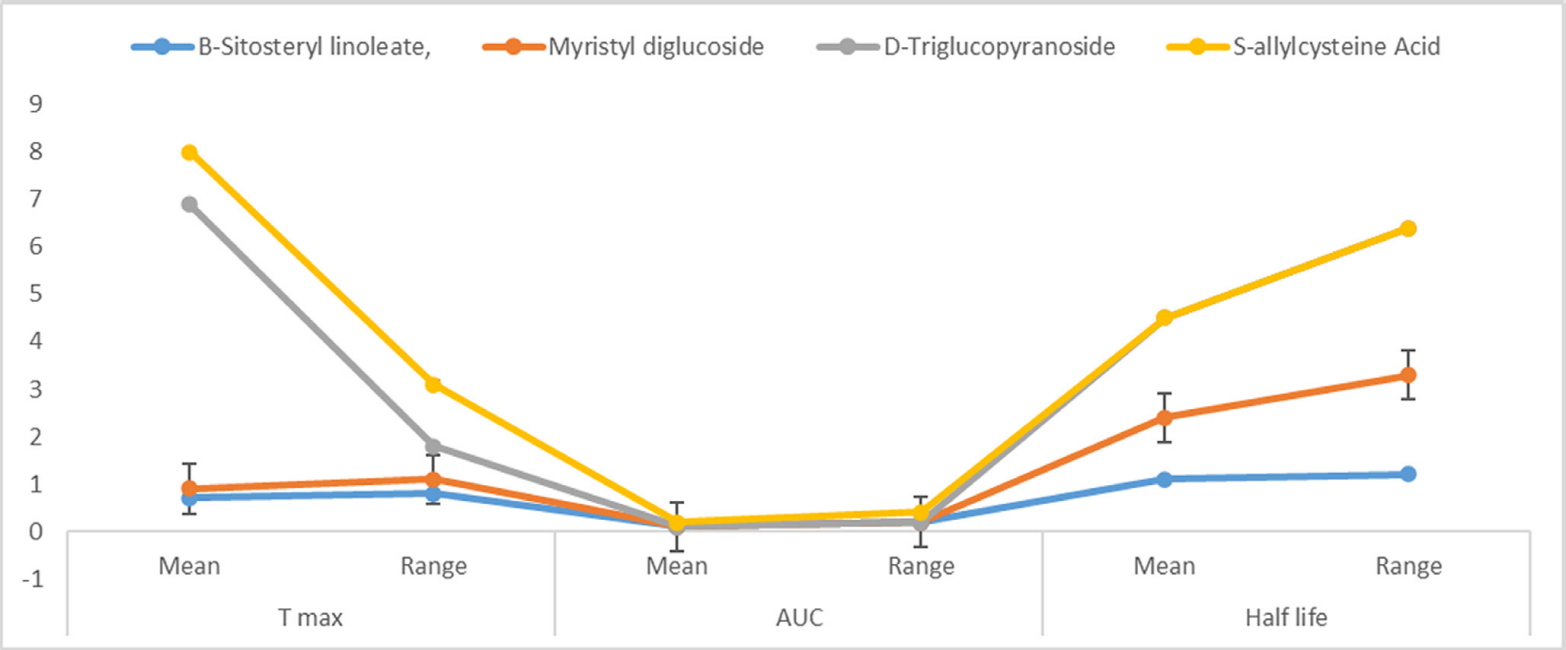
Drugs design of antibiotic compounds its changes.

The entire formulation of s-allylcysteine acid, D-Triglucopyranoside, Myristyl diglucoside, and B-Sitosteryl linoleate was investigated using pharmacokinetic modelling. Table 8 shows the results of the modeling, including the active variable of the whole maximal range of activity under plasma concentrations, T max times, area under plasma concentration (AUC). The T-max times with mean values (0.2–1.1) and ranges of B-Sitosteryl linoleate and S-allylcysteine acid in leave extracts of adhatoda vasica were the responses of all four compounds with their mean values and ranges of three active factor However, the half-lives over all three B-Sitosteryl linoleate, Myristyl diglucoside and D-Triglucopyranoside molecules were the same (1.1–3.1). One compound’s half-life was missing. The range of T-max times for mean values was (0.2–1.1), while the range for allfour compounds (0.3–1.2). In leave extracts of Calotropis procera, the area under plasma concentrations was (0.1, 0.2) in two components of B-Sitosteryl linoleate and S- allylcysteine acid. However, the half-lives of all three B-Sitosteryl linoleate, Myristyl diglucoside, and D-Triglucopyranoside molecules were the same (1.1–4.1). Fig. 8 shown the compounds dose with the maximum time.

**Table 8:** Drugs formulations and compartment modelling in pharmacokinetic studies of chemicals for suitable 528 medicines.

Antibiotic Bioactive compounds	*Adhatoda vasica*						*Calotropis procera*					
	T max		AUC		Half life		T max		AUC		Half life	
	Mean	Range	Mean	Range	Mean	Range	Mean	Range	Mean	Range	Mean	Range
B-Sitosteryl linoleate,	0.7	0.8	0.1	0.2	1.1	1.2	0.5	0.9	0.1	0.2	1.1	2.2
Myristyl diglucoside	0.2	0.3	–	–	1.3	2.1	0.2	0.3	–	–	1.3	3.1
D-Triglucopyranoside	6.0	0.7	–	–	2.1	3.1	2.0	0.7	–	–	2.1	4.1
S-allylcysteine Acid	1.1	1.3	0.1	0.2	–	–	2.0	0.7	–	–	2.1	4.1

T max, total maximum AUC, area under plasma concentration, Half-life of compounds, Mean of three determinations ± SD of herbal plants compounds as used in medicines.

### 3.9 Pharmacokinetic modelling acid dissociation constant (pKa), solubility and absorption capacity

**Fig. 9:**
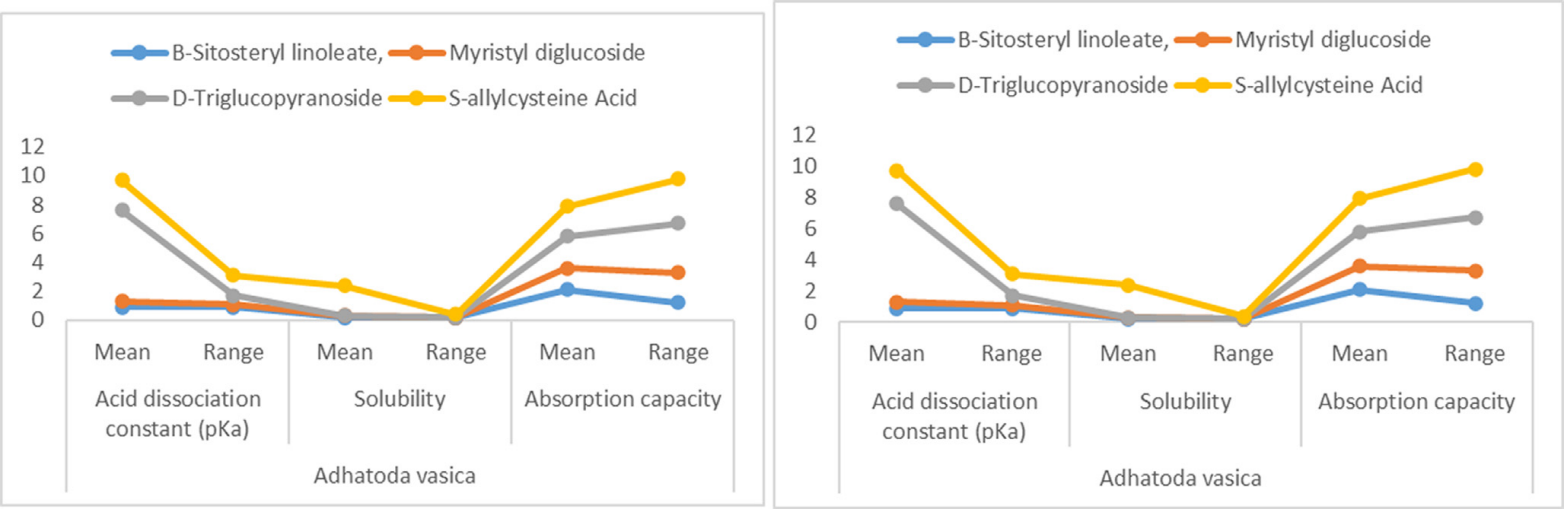
Drugs design of antibiotic compounds its changes.

The pharmacokinetic modelling was applied to the entire medication compositionsof these compounds (D-Triglucopyranoside, Myristyl diglucoside, s-allylcysteine acid and B-Sitosteryl linoleate) were studied as shown in Table 9 and Fig. 9. The modelling including the acid dissociation constant (pKa), solubility and compounds' reactions were documented, as well as the manner of absorption capacity of all four compounds shown in Table 9. Acid dissociation constant (pKa), with averages (0.4–2.1) and the outputs of all constituents including their overall mean as well as ranges of 3 major variables in *A. vasica* leaf extracts were S-allylcysteine Acid and B-Sitosteryl linoleate. However, the absorption capacity of D-Triglucopyranoside, Myristyl diglucoside, B-Sitosteryl linoleate were between (1.1–3.4). The average value of acid dissociation constant (pKa) lied between (0.2–0.9) as well as the average range of all chemicals was (0.1–2.1). In different substances of B-Sitosteryl linoleate and S-allylcysteine acid, the solubility was (0.1, 0.2) in extract solution of *C. procera*. Furthermore, all three Myristyl diglucoside, D-Triglucopyranoside B-Sitosteryl linoleate, compounds had the same absorption capability (2.1–4.5).

**Table 9:** Design and mode of action of compounds responses under Pharmacokinetics with bioability of compounds changes

Antibiotic Bioactive compounds	*Adhatoda vasica*						*Calotropis procera*					
	Acid dissociation constant (pKa)		Solubility		Absorption capacity		Acid dissociation constant (pKa)		Solubility		Absorption capacity	
	Mean	Range	Mean	Range	Mean	Range	Mean	Range	Mean	Range	Mean	Range
B-Sitosteryl linoleate,	0.9	0.9	0.2	0.2	2.1	1.2	0.5	0.9	0.1	0.2	2.1	2.2
Myristyl diglucoside	0.4	0.2	0.1	–	1.5	2.1	0.2	0.3	0.1	–	2.3	3.1
D-Triglucopyranoside	6.3	0.6	–	–	2.2	3.4	2.0	0.7		–	3.1	4.1
S-allylcysteine Acid	2.1	1.4	2.1	0.2	2.1	3.1	2.0	0.7	2.1	0.2	2.1	4.7

Mean of three determinations ± SD of herbal plants compounds as used in medicines.

### 3.10 New pharmacological responses to extracts on Lactate Dehydrogenase (LD) performance

**Fig. 10:**
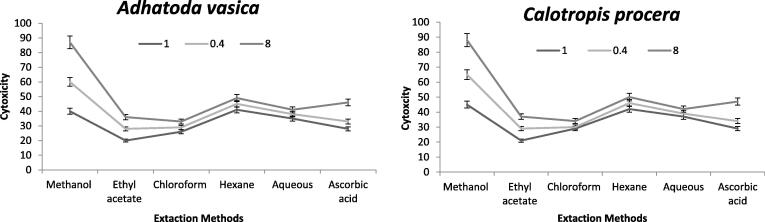
Potential cytotoxicity level of two herbal species for controlling of toxins in different at Bacterial strains. Each vertical bar represents mean of three replications.

Only methanol extracts of both plants were used in the cytotoxicity assay and thre concentrations were used (1, 0.4 and 0. 8). In menthol solution, a higher cytotoxic value for 0.8% accumulation was reported to be 87 μg/ml. In *A. vasica* leave extract, the maximum potency was seen in the following order: ethyl acetate > chloroform > hexane > aqueous > ascorbic acid. It seems to be worth mentioning that the greater cytotoxicity test only assesses 0.8% solution concentrations rather than 0.4 and 1% solution concentrations and also that the maximal cytotoxicity level from separated components of *C. procera* leaf extracts was 9087 μg/ml at a concentration of 1% in standard methanol extracted solutions. The cytotoxicity solutions (0.4 and 0.8%) on the other hand, was shown to be lower. Fig. 10 presented the cytotoxicity results. It was 1% from both herbal plants, according to standardization of concentration for cytotoxicity tests.

### 3.11 Bio-efficacy of bioactive compounds and lipid peroxidation in vitro for drug development

**Fig. 11:**
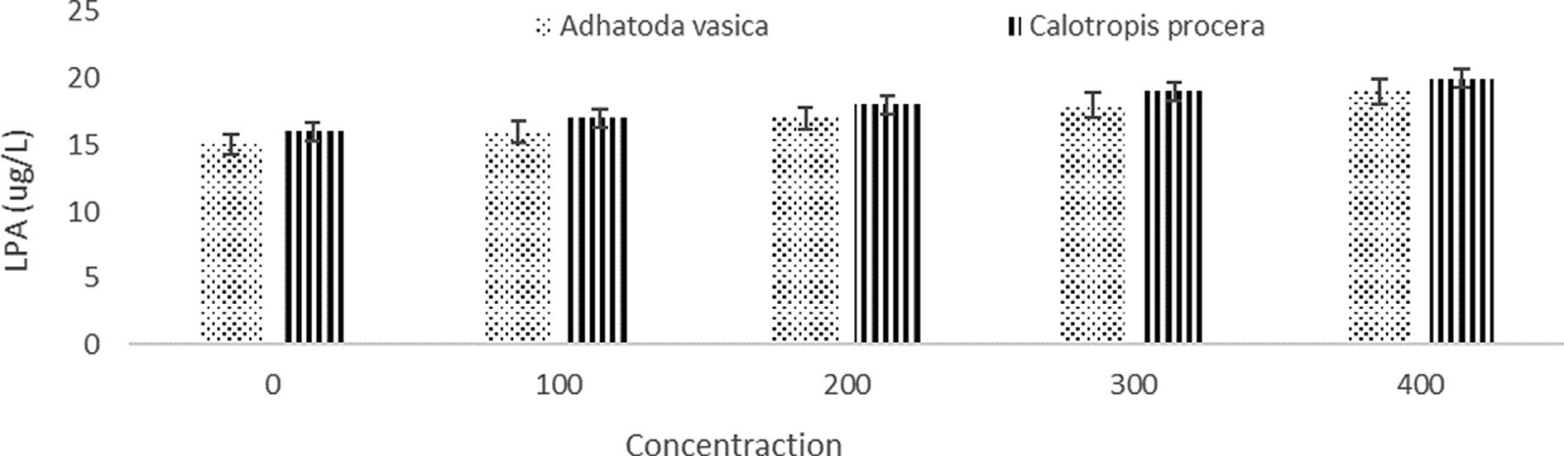
Lactate dehydrogenase (LD) activity assay from *A. vasica* and C.proce.

Both plant leaf extracts depicted in Fig. 11 were used in the in vitro lipid peroxidation tests. The LAP (20 μg/l) involvement is higher in the concentration of 400% as compared with the other concentrations of 100, 200 and 300% of solutions were acquired in the extracts of *A. vasica* and *C. procera* showed similar patterns.

## 4 Discussion

Currently the world is dealing with a slew of contagious diseases, as well as the health consequences of chronic diseases (Cakir et al., 2004). Chemists are constantly working to identify new antibacterial resources from herbal plants for the successful treatment of a variety of ailments (Pragasam and Rasool, 2013). Some scientists reported that bioactive chemicals were resistant to infectious and bacterial disorders (Perdicaris et al., 2013). The present study reveals that methanol extracts we successful in isolating pure chemicals for extraction, which could be due to their solubility in methanol solution, as previously described in many studies (Cakir et al., 2004, Rastogi et al., 1994, Williams and Abdi, 2010, Khan et al., 2012). The findings revealed that isolated chemicals have a significant antibacterial capacity at the cytoplasmic membrane. These acids, isomartynoside and acetonide, are found in plants and have the potential to reduce bacterial strains, particularly for waterborne infections (Khan et al., 2012). The current findings support prior observations by (Asahina and Shibata, 1954, Shan et al., 2007), they found that *trans*-acetonide serves as an antibacterial representative for both strains of bacteria (Contreras et al., 2015). Likewise, extensive clinical experiments studies show certain compounds (acids) are beneficial in the treatment of a variety of human disorders (Cakir et al., 2004, Khan et al., 2012). The discovery of high resistance chemicals from both plants demonstrated their useful effect on medication formulation (Lambert et al., 2001). Isomartynoside has conjugated capabilities with polyamines, polysaccharides, lipids and mono- and oligosaccharides making it a helpful part of Chinese medicine and herbal products (Shearer et al., 20032003). It has antioxidant, antibacterial, anti-cancer, anti-thrombosis and anti-inflammatory properties (Nikaido et al., 1998). Correspondingly, 2,4-Chebulic-beta-D-glucopyranose acid and S-allylcysteine acid demonstrated better impedance to many bacterial infections. The current study's findings (Table 3) are comparable to those of (Shan et al., 2007), who stated that novel antimicrobial drugs should be investigated in the control ofvarious diseases (Pragasam and Rasool, 2013). Both herbaceous species had the highest levels of FRAP expression in their leaves in comparison with ascorbic acid, explained by this experiment for first time. Phytochemicals are shown to improve resistance by modulating both adaptive and typicalimmune responses. It was significant to observe that all bacterial strains exhibited remarkable vulnerability for regulation of antibacterial properties in nearly in all extract and this was consistent with earlier reports that herbal phytochemicals as natural antioxidants and immune-modulators affected both positive and negative bacteria (Shan et al., 2007). For a range of disorders, immunomodulation with medicinal plants could be a viable alternative to traditional chemotherapy. The MCB in methanol extracts is decreased because of cell membrane blockage (Contreras et al., 2015, Nikaido et al., 1998, Deans and Ritchie, 1987, Doerrler et al., 2001). Diverse sample solutions contain distinct antioxidants which employ a variety of different mechanisms to combat certain germs (Contreras et al., 2015, Zink, 1997). It was discovered that all extracts had the greatest antibacterial action against both bacterial strains (positive and negative), which was linked to chemicals found in leaf extracts that function as antioxidants. The antioxidant effects are attributed to the several significant phytochemical substances found in these two species, which are known assist the plant's bioactive qualities. The consequences of the LOQ and LOD tests revealed that the approach used to quantify and qualify the bioactive components of both plant species are precise and dependable.

## 5 Conclusions

As in the research, the antibacterial actions of some extracts from Adhatoda vasica and Calotropis procera showed that both plants have the most powerful phytochemicals, which have been revealed to help with oxidative stress and other ailments. Myristyl diglucoside D-Triglucopyranoside, S-allylcysteine acid, B-Sitosteryl linoleate) were identified in *A. vasica* and *C. procera* samples and investigated in six different solvents.D-Triglucopyranoside (13.81 ± 0.48%), Myristyl diglucoside (11.81 ± 0.41%), B-Sitosteryl linoleate (12.81 ± 0.48%), and S-allylcysteine acids (14.81 ± 0.31%) were detected in large concentrations in methanol extract. The MBC for E-coil was 335 g/ml and K. numoneae was 272 g/ml, from *A. vasica* isolated leaves solutions. Methanol extraction of Adhatoda vasica yielded the highest ferric lowering antioxidant potential. 12.81 ± 0.40 mg TE100 g^−1^, while ascorbic acid (2.910.03 TE100 g^−1^). The outcome revealed that extracts (methanol) had a stronger concentration of phytochemicals than other extraction techniques. Supercritical fluid extraction is the best method for the extraction of compounds. The design of compounds has approved the quantitation of cells montoinered in drug design and developmental process. They can be used in place of or in addition to traditional chemotherapy for a variety of conditions, particularly whenever the host's defensive system needs must have been activated due to a compromised immune response or when targeted immunosuppression is needed in situations like autoimmune disorders. Both plants have higher antioxidants and antibacterial activity. The complete pharmacokinetic and drug design prove an efficient range of compounds and prove to be used in future medicines. Similar compounds have the potential of remembrances stability and are less infectious. It was concluded that the method of extraction was accurate, reliable and safe for pure compounds extraction. The isolated chemicals, according to the study, can be employed as the finest medical resource for treating both bacterial variants (positive and negative gram bacteria) in humans. These chemicals were strongly employed for the treatment of several illnesses conditions, according to pharmacokinetic and bioavailability data.

## Ethics approval and consent to participate

The human bacterial strains are obtained in hospital with codes are mentioned.

## Consent for publication

Not applicable.

## Availability of data and materials

Yes data and materials are available on request and demand.

## Authors' contributions

WA contributed in collecting plant sample, identification and herbarium confection. Conceived and designer the experiments: WA, RA. Performed the experiments RA, AQ, AM, ML. Analyzed the data: WA, AQ, MA, AM, SMK. Wrote the paper: WA, RA, ML, and SMK. All the authors have read the final manuscript and approved the submission.

## Funding

This study was supported by Taif University Researchers Supporting Project number (TURSP-2020/132), Taif University, Taif, Saudi Arabia.

## Declaration of Competing Interest

The authors declare that they have no known competing financial interests or personal relationships that could have appeared to influence the work reported in this paper.
